# A meta-analysis of the prevalence of attention deficit hyperactivity disorder in incarcerated populations

**DOI:** 10.1017/S0033291714000762

**Published:** 2014-04-07

**Authors:** S. Young, D. Moss, O. Sedgwick, M. Fridman, P. Hodgkins

**Affiliations:** 1 Centre for Mental Health, Division of Brain Sciences, Department of Medicine, Imperial College London, UK; 2 Broadmoor Hospital, West London Mental Health Trust, London, UK; 3 Caudex Medical, Oxford, UK; 4 Department of Psychology, Institute of Psychiatry, King's College London, UK; 5 AMF Consulting, Inc., Los Angeles, CA, USA; 6 Global HEOR, Vertex Pharmaceuticals, Boston, MA, USA

**Keywords:** ADHD, crime, diagnosis, incarcerated, offender, prevalence, prison

## Abstract

**Background:**

Studies report the variable prevalence of attention deficit hyperactivity disorder (ADHD) in incarcerated populations. The aim of this meta-analysis was to determine the prevalence of ADHD in these populations.

**Method:**

Primary research studies reporting the prevalence (lifetime/current) of ADHD in incarcerated populations were identified. The meta-analysis used a mixed log-binomial model, including fixed effects for each covariate and a random study effect, to estimate the significance of various risk factors.

**Results:**

Forty-two studies were included in the analysis. ADHD prevalence was higher with screening diagnoses *versus* diagnostic interview (and with retrospective youth diagnoses *versus* current diagnoses). Using diagnostic interview data, the estimated prevalence was 25.5% and there were no significant differences for gender and age. Significant country differences were noted.

**Conclusions:**

Compared with published general population prevalence, there is a fivefold increase in prevalence of ADHD in youth prison populations (30.1%) and a 10-fold increase in adult prison populations (26.2%).

## Introduction

Attention deficit hyperactivity disorder (ADHD) is defined in DSM-IV (APA, 1994) and ICD-10 (WHO, 1992) as high levels of hyperactive, impulsive and/or inattentive behaviours beginning in early childhood. A level of impairment in at least two areas of life needs to be evident for at least 6 months' duration for a diagnosis of ADHD to be made. ADHD is a common disorder that often persists into adulthood (Faraone *et al.* 2006), affecting 3–7% of youths (Asherson, 2004; Asherson *et al.* 2005; Polanczyk *et al.* 2007; Willcutt, 2012) and 1–5% of adults (Asherson, 2004; Asherson *et al.* 2005, 2012; Kessler *et al.* 2006; Simon *et al.* 2009; Willcutt, 2012).

There is no generally accepted estimate of the prevalence of ADHD in incarcerated populations; research studies have consistently reported a disproportionately higher rate of individuals with ADHD in the criminal justice system compared with general population prevalence. These individuals, who are often untreated, seem to have greater judicial contact and pose higher risk within the criminal justice system. Compared with other offenders, they present with younger age at first contact, greater recidivism and institutional behaviour disturbance (Young *et al.* 2003, 2009*b*, 2011). A Scottish prison study found that inmates with ADHD symptoms were involved in up to eight times more institutional aggression; this strong association was maintained even when controlling for antisocial personality characteristics, with aggressive incidents being six times greater compared with non-ADHD peers (Young *et al.* 2009*b*). These problems are likely to be associated with underlying deficits in executive dysfunction (Young *et al.* 2007; Bramham *et al.* 2009; Rose *et al.* 2009); in particular, behavioural disinhibition and emotional dysregulation (Gudjonsson *et al.* 2009, 2012).

However, prevalence varies widely among studies and methodologies differ. Hence, this study reviewed the data obtained from a systematic literature search performed to ascertain the lifetime/current prevalence of ADHD in prison populations in both youths and adults. The study aimed to specify (1) the prevalence of ADHD in incarcerated youth and adult offenders, including differences by gender; (2) prevalence from studies applying screening tools and diagnostic clinical interviews; (3) differences in prevalence obtained from retrospective diagnoses from adults and youth prevalence obtained using a current diagnoses methodology; and (4) geographical differences.

## Method

### Eligibility criteria

The systematic review was performed in accordance with the Preferred Reporting Items for Systematic reviews and Meta-Analyses (PRISMA; Liberati *et al.* 2009) guidelines. Initial searches were carried out in a variety of databases and websites to gain an understanding of the amount of information available. Reports published since 1980 and in English were included.

### Data sources

Searches were conducted in OvidSP Medline (1948 to present) and EMBASE (1988 to present segment), Datastar PsycINFO (unrestricted) and Social SciSearch (1972 to date; limited to English and added since 1 January 1980), including the literature published between 1 January 1980 and 3 May 2011; a citations update was performed on 21 June 2011 and extended to 31 August 2012 in October 2012.

### Search

Search terms were developed, refined and tested for relevance by cross-checking results against a list of known relevant articles provided by the lead author. The following descriptors were used in OvidSP EMBASE (1988 to present segment): ADHD; attention deficit disorder; [EMTREE] crime; criminals; criminology; criminal behaviour; criminal justice; criminal law; court; criminal psychology; delinquency; juvenile delinquency; gang; legal evidence; legal procedure; police; legal liability; mandatory programs; violence; prisons; prisoner; probation; law enforcement; recidivism; jurisprudence; punishment; offender; drug abuse; drug misuse.

### Study selection

Articles obtained from the final searches were first de-duplicated, then an inclusion/exclusion process was undertaken based on the following exclusion criteria: non-English language articles, articles published before 1980, animal studies, articles that were not peer reviewed (e.g. theses), and articles that obviously did not hold relevance (e.g. they did not focus on ADHD or crime, or they focused on substance misuse but not from the criminology perspective). Review articles pre-2006 were excluded, and post-2006 reviews were kept with the sole purpose to examine bibliographies to check for any other articles not identified in the search; these review articles were not included in the final prevalence calculation; only primary research articles were included. Articles with no abstract (including initial PsycINFO and Social SciSearch search outputs) were also excluded unless the title or other information (e.g. key terms) suggested they may hold relevance. The inclusion/exclusion review was first completed based on title/abstract/key words by four researchers, and if the relevance of an article was unclear, the full text was retrieved before a final decision was made. Once the initial bulk of the inclusion/exclusion process was completed, abstracts were reviewed independently by the lead author before full-text articles were retrieved. Any uncertainties over including/excluding articles were discussed among the researchers and a final decision was made once the full-text articles were reviewed.

The full texts of included articles were retrieved for detailed evaluation against eligibility criteria. Only the publications that focused on incarcerated/prison populations and those that reported ADHD prevalence were selected. Studies that reported results on mixed gender populations were excluded as we aimed to separate the gender effect in modelling.

### Data collection process

A data extraction sheet was developed in Microsoft Excel and pilot tested on 13 randomly selected studies and refined accordingly. The publications were divided among four researchers (including two authors: D.M. and O.S.) who performed the data extraction independently. Data were reviewed for consistency and any queries were resolved by discussion among the researchers and the lead author. The lead author also made the final decision whether to include/exclude data by reviewing the identified publications.

The authors of 11 studies were contacted directly in personal communications to ascertain missing information relating to age of the study population (Black *et al.* 2004; Sanz-Garcia *et al.* 2010*a*, *b*), gender (Vitelli, 1996), method of assessment of ADHD (Gordon & Moore, 2005), study population (Cahill *et al.* 2012; Colins *et al.* 2012) and verification of the incarcerated status of the sample (Langevin, 2003; Rosler *et al.* 2009; Retz & Rosler, 2010; Lindsay *et al.* 2012). All responded with additional information that had not been included in the original publication and, if meeting inclusion criteria, was included in this meta-analysis (indicated in Supplementary Tables S1 and S2).

Some cohorts of prison populations were published more than once. To avoid double counting data, multiple reports of the same cohort were pieced together by juxtaposing author names, treatment comparisons, sample sizes and outcomes. An exception to this rule occurred in relation to the Young *et al*. (2010*a*, 2011) reports; whereas Young *et al.* (2010*a*) used diagnostic interviews, Young *et al.* (2011) used screening data to provide ADHD prevalence. In the interest of obtaining meaningful data on diagnostic differences in ADHD, these reports were considered as separate studies. In addition, following author communication, more recent data from Cahill *et al.* (2012) were included rather than data from an earlier publication of the same population (Coolidge *et al.* 2009).

### Data items

For each included study, data were extracted into a data set containing: (1) study location (country of origin), (2) study sample size, (3) study population (including age and gender), (4) diagnostic criteria (screening or diagnostic interview), (5) study design (retrospective, that is adult studies reporting a prevalence of childhood ADHD, or current diagnoses), (6) ADHD prevalence and (7) treatment (including prison management). We have used the term ‘prison’ but included all individuals who were incarcerated, jailed or imprisoned, or similar descriptors. For the analysis, studies were grouped by age for youth and adult offenders (see Supplementary Tables S1 and S2). It was noted that there was no clear definition across publications on the age of an adult *versus* a youth. For the purposes of this meta-analysis, we designated 18 years to be the cut-off point for a youth becoming an adult (i.e. youths were ⩽18 years and adults were >18 years); papers reporting youth data often cited 18 years as the upper limit of the age range of participants. In those papers where an age range was given that spanned this cut-off point (e.g. 15–28 years), the mean (or median if mean was not provided) was used to define whether the study population should be listed as ‘youth’ or ‘adult’. For studies reporting on (1) both genders separately, (2) both diagnostic criteria or (3) both study designs, information was recorded as two separate observations (‘study strata’) linked by the study number. None of the studies reported separate results on more than one of these three characteristics and none reported on both age groups.

For a diagnosis of ADHD to be given in adulthood, clinically significant symptoms must have been present in childhood. Therefore, when assessing adults for ADHD, it is necessary to establish whether they meet criteria for childhood ADHD, requiring them to retrospectively report on their symptoms (if a diagnosis did not already exist). Some adult studies (*n* = 13) included in this meta-analysis also reported a prevalence of childhood ADHD for the cohort (referred to as a retrospective diagnosis in this study).

### Quality control

To ascertain the validity of eligible publications, each researcher checked and independently reviewed a random sample of each others' papers for data extraction and interpretation consistency. Disagreements were resolved by reviewing the data source and by discussion between the three reviewers (one of whom is an author).

### Statistical methods

Summary prevalence estimates were calculated for the meta-analysis. Observed prevalence per study stratum was reported by age and gender groups. Model-predicted prevalence was reported by the various model covariates together with their corresponding 95% confidence intervals (CIs).

A mixed log-binomial model was fitted for the observed prevalence in the study strata to estimate the significance of the various risk factors (model 1). Covariates collected for predicting ADHD prevalence included gender, age group, diagnostic method, study design and country. The model included fixed effects for each of the covariates and a random study effect, and can be defined as follows: 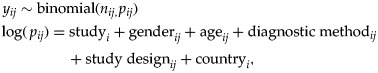 where *y*_*ij*_ is the number of patients with the response, *p*_*ij*_ is the ADHD prevalence and *n*_*ij*_ is the total number of inmates included in study *i* and on stratum *j*. As implemented here, the model made the following assumptions:

(1) study_*i*_ represented a random study effect, assumed to follow a normal (0, *σ*^2^) distribution. The study effect was considered to be the within-study logarithmic baseline prevalence, thus eliminating the study-specific effect from the other comparison estimates.
(2) gender_*ij*_ represented the adjusted gender effect common to all patients in study *i* and stratum *j* on the logarithm of prevalence. Similarly for age_*ij*_, diagnostic method_*ij*_, study design_*ij*_ and country_*i*_. Countries were also grouped into regions in an attempt to obtain more stable estimates (North America, Europe and Other).

Given the observed level of interaction between gender, age group and diagnostic method, these three covariates were fully interacted in the model and were represented by eight indicator variables (one omitted as a reference group). Other interactions were also tested for significance. Marginal estimates of prevalence (least-square means) for each of the covariates (e.g. males, adults, etc.) and for their combinations were calculated by applying the inverse logarithmic transformation to model predictors. To estimate the sensitivity of results to the diagnostic method, the model was refitted to a subset of data using diagnostic interview strata only (model 2). For the sensitivity analysis model (model 2), the aforementioned eight indicator variables for the interaction of gender, age group and diagnostic method were collapsed to four to capture the interaction of age-by-gender group.

The models were fitted adopting a frequentist estimation approach using PROC GLIMMIX in SAS version 9.2 (SAS Institute, USA).

## Results

**Fig. 1.**
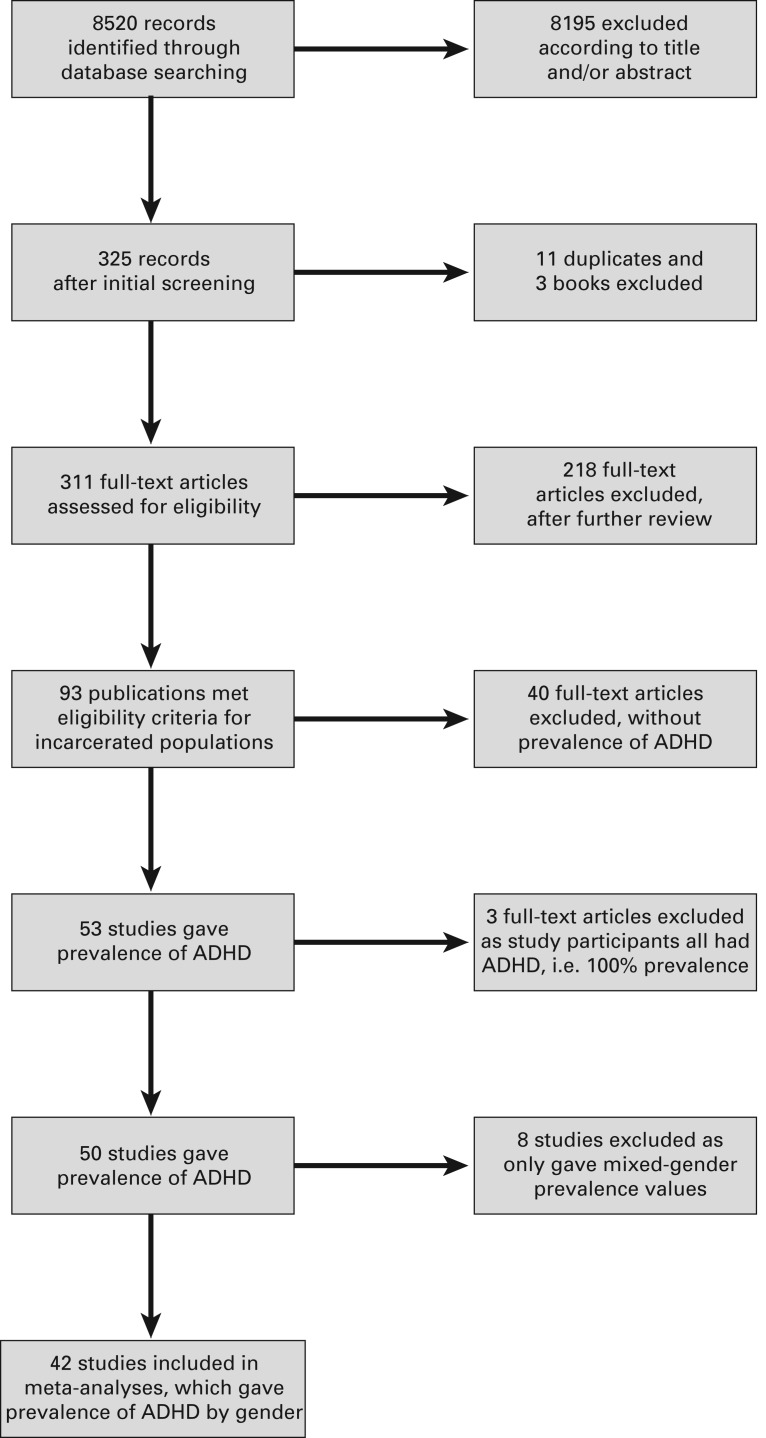
Flow diagram of the manual screening process for eligible literature inclusion. ADHD, attention deficit hyperactivity disorder.

In total, 8520 publications were identified following the OvidSP Medline, EMBASE, Datastar, PsycINFO and Social SciSearch database search. Publications not specifically relating to ADHD and criminality and duplicates between databases were excluded electronically, leaving 325. Once further duplicates had been identified manually, 311 publications remained. A further 218 publications were excluded after further review as they did not report on samples composed entirely of incarcerated/prison populations. Of the 93 articles remaining, a further 40 articles were excluded as they did not provide prevalence data on ADHD; 53 publications included ADHD prevalence data. Three studies (Young *et al.* 2009*a*; Ginsberg & Lindefors, 2012; Tidefors & Strand, 2012) were excluded as they used samples composed solely of participants who had ADHD, hence giving a prevalence rate of 100%. A further eight studies were excluded as they only provided mixed-gender prevalence (Milin *et al.* 1991; Fulwiler *et al.* 1997; Rasmussen *et al.* 2000, 2001; Black *et al.* 2004, 2010; Anckarsater *et al.* 2007; Einat & Einat, 2008; Gudjonsson *et al.* 2009), leaving 42 studies included in the meta-analysis, reporting prevalence by gender (Fig. 1).

### Prevalence

**Fig. 2.**
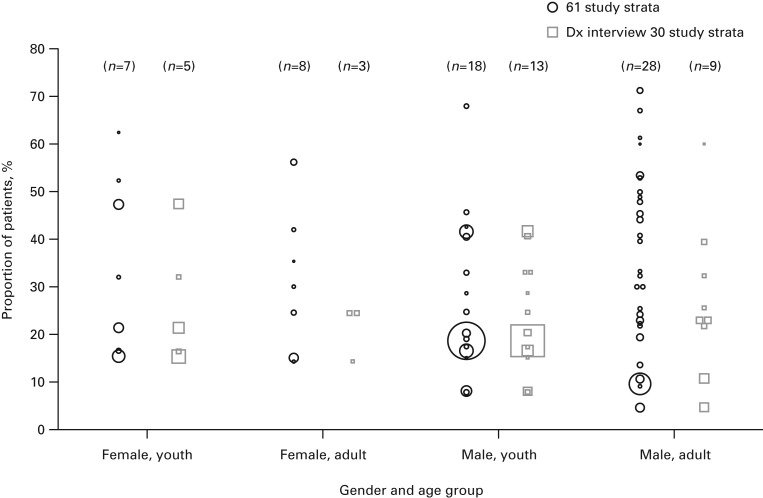
Observed attention deficit hyperactivity disorder prevalence per study stratum by age group (youths ⩽18 years and adults >18 years) and gender showing all data (42 studies, 61 strata) and interview diagnosis (Dx) strata (21 studies, 30 strata). Each bubble/square in the plot represents a study stratum. Bubble/square areas are proportional to stratum sample size. The numbers of study strata per column are shown in parentheses.

**Fig. 3.**
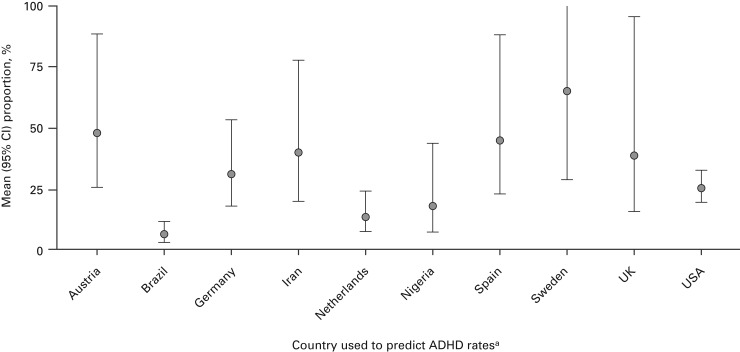
Model-predicted attention deficit hyperactivity disorder (ADHD) prevalence by country (model 2: diagnostic interview only). ^a^ Upper bounds of 95% confidence intervals (CIs) are truncated to 1.0.

**Fig. 4.**
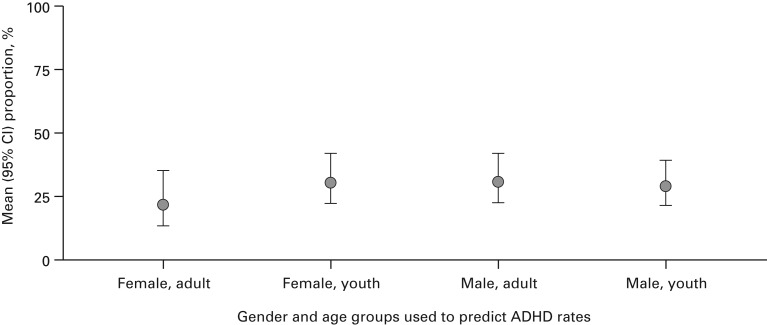
Model-predicted attention deficit hyperactivity disorder (ADHD) prevalence by gender group and age groups (youths ⩽18 years and adults >18 years; model 2: diagnostic interview only). CI, Confidence interval.

Observed ADHD prevalence per study stratum by age group (youths ⩽18 years and adults >18 years) and gender showing all data (42 studies, 61 strata) and interview diagnosis strata (21 studies, 30 strata) are presented in Fig. 2. Bubble plots show the ADHD prevalence per study stratum for the full sample and the subset of study strata where a diagnostic interview was administered. The plots demonstrate that some of the high prevalence outliers are study strata where screening was used for ADHD diagnosis. Thus, all other data used diagnostic data only. No other differences were observed between the gender and age groups. Table 1 and Figs 3 and 4 (and Supplementary Figs S1 and S2) summarize the results obtained from model 1 (all data, that is both screening and diagnostic interview) and model 2 (diagnostic interview subset only). There were no significant differences between genders in both models.

**Table 1.** Summary of prevalence results (%) from fitted models (youths ⩽18 years and adults >18 years)

Covariate	Model 1: All data	Model 2: Diagnostic interview subset
Gender × Age group × Diagnostic method		
F, Adult, Interview (*n* = 3)	17.8	n.a.
F, Adult, Screen (*n* = 5)	37.8	
F, Child/Adolescent, Interview (*n* = 5)	31.3	
F, Child/Adolescent, Screen (*n* = 2)	66.5	
M, Adult, Interview (*n* = 9)	25.3	
M, Adult, Screen (*n* = 19)	30.7	
M, Child/Adolescent, Interview (*n* = 13)	30.0	
M, Child/Adolescent, Screen (*n* = 5)	45.6	
*p* value	0.006	
Gender × Age group		
F, Adult (*n* = 3)	n.a.	22.1
F, Child/Adolescent (*n* = 5)		30.8
M, Adult (*n* = 9)		31.2
M, Child/Adolescent (*n* = 13)		29.5
*p* value		0.242
Study design (*n*_1_ = 61, *n*_2_ = 30)		
Current diagnoses	23.4	21.7
Retrospective child diagnoses	47.1	36.4
*p* value	<0.0001	<0.001
Country^a^		
Austria (*n*_1_ = 2, *n*_2_ = 2)	60.7	47.8
Brazil (*n*_1_ = 2, *n*_2_ = 2)	9.4	6.6
Canada (*n*_1_ = 1, *n*_2_ = 0)	49.1	
Germany (*n*_1_ = 5, *n*_2_ = 2)	49.3	31.4
Greece (*n*_1_ = 1, *n*_2_ = 0)	45.6	
Iceland (*n*_1_ = 4, *n*_2_ = 0)	39.2	
Iran (*n*_1_ = 1, *n*_2_ = 1)	51.0	40.1
Ireland (*n*_1_ = 1, *n*_2_ = 0)	13.4	
South Korea (*n*_1_ = 2, *n*_2_ = 0)	35.5	
Netherlands (*n*_1_ = 2, *n*_2_ = 2)	18.0	14.2
Nigeria (*n*_1_ = 1, *n*_2_ = 1)	22.8	18.1
Spain (*n*_1_ = 3, *n*_2_ = 1)	50.4	45.5
Sweden (*n*_1_ = 8, *n*_2_ = 1)	50.1	65.2
UK (*n*_1_ = 4, *n*_2_ = 1)	31.4	39.4
USA (*n*_1_ = 24, *n*_2_ = 17)	33.8	25.7
*p* value	<0.002	<0.0001

F, Female; M, male; n.a., not applicable.

a The number of study strata included are listed in parentheses for each covariate (number of strata in models 1 and 2 are provided for covariates in both models, respectively). The numbers of studies in each country are: Austria (*n* = 1), Brazil (*n* = 1), Canada (*n* = 1), Germany (*n* = 3), Greece (*n* = 1), Iceland (*n* = 2), Iran (*n* = 1), Ireland (*n* = 1), South Korea (*n* = 1), Netherlands (*n* = 2), Nigeria (*n* = 1), Spain (*n* = 2), Sweden (*n* = 6), UK (*n* = 3), USA (*n* = 16). All studies are listed in Supplementary Tables S1 and S2.

Overall, the observed ADHD prevalence in prison populations in all 42 studies (61 study strata) was 21.3% (total number of inmates was 26641, of whom 5677 were reported to be diagnosed with ADHD). The observed ADHD prevalence in prison populations for inmates with an interview diagnosis (21 studies, 30 study strata) was 20.5% (total number of inmates was 19575, of whom 4008 were reported to be diagnosed with ADHD).

#### Screening versus diagnostic clinical interview methodology

Studies using screening for diagnosis had a significantly higher estimated ADHD prevalence of 43.3% (95% CI 33.2–56.4) compared with 25.5% (95% CI 20.0–32.4) obtained from the subset of studies using a diagnostic clinical interview (*p* = 0.001). This suggested a high rate of false positives was being identified by screening tools, and thus subsequent reported analysis used prevalence obtained only from diagnostic clinical interview data (i.e. model 2).

#### Retrospective versus current youth diagnoses methodology

ADHD prevalence was estimated to be significantly lower (*p* < 0.001) in studies with current diagnoses; ADHD prevalence for current diagnoses was 21.7% (95% CI 17.5–26.9), compared with 36.4% (95% CI 25.6–51.8) for retrospective diagnoses.

#### Geographical differences

There were significant differences in ADHD prevalence estimated across countries (*p* < 0.0001). ADHD prevalence ranged from 6.6% in Brazil (Ponde *et al.* 2011) to 65.2% in Sweden (Table 1 and Fig. 2; model 2 diagnostic interview). When aggregated into three regions (North America, Europe and Other countries), differences were not significant when fitting models similar to model 2, replacing countries with regions (*p* = 0.240); Europe had the highest estimated prevalence (32.1%), followed by North America (26.9%) and Other countries (17.6%).

#### Males versus females

Prevalence estimates for males with ADHD were not significantly different from estimates for females with ADHD: 30.3% (95% CI 23.9–38.4) *versus* 26.1% (95% CI 19.3–35.2).

#### Youths versus adults

Youth prevalence estimates were not significantly different from estimates for adults: 30.1% (95% CI 22.1–41.1) *versus* 26.2% (95% CI 18.4–37.5).

#### Gender-by-age interaction

Gender-by-age (Table 1 and Fig. 4) comparisons reported that female adults had the lowest predicted ADHD prevalence: 22.1% (95% CI 13.6–35.8). By contrast, the prevalence estimate for male adults was 31.2% (95% CI 22.7–42.9). Female and male youth estimates were 30.8% (95% CI 22.4–42.2) and 29.5% (95% CI 21.6–40.1), respectively.

There were no significant differences found within the clinical interview diagnostic groups among the four gender-by-age groups.

## Discussion

### Prevalence

This review presents a meta-analysis of 42 studies conducted in 15 countries that provides a reliable estimate of 25.5% as the prevalence of ADHD in incarcerated populations from studies using diagnostic clinical interview. Heterogeneity is likely to be present in most meta-analyses as individual studies with the ‘same’ population and study characteristics are never identical with respect to both measured and unmeasured factors, which can lead to differences in outcome. For this reason, models that allow for heterogeneity are recommended (DerSimonian & Laird, 1986; van Houwelingen *et al.* 2002). We fitted models that assumed a fixed effect (equal across studies) of measurable covariates, such as gender or age group, on ADHD prevalence and a random effect for study that allowed for the same types of subjects (e.g. male adult with a diagnostic interview and current diagnoses design) to have different prevalence in different studies, and also provided for a mechanism to allow prevalence correlation in the same study strata (intra-cluster correlation).

In recent years, a high prevalence of psychopathology (depression, psychotic illness, ADHD, conduct disorder) has been recognized in prison inmates (Fazel *et al.* 2008*a*, *b*; Bradley, 2009), and although more comprehensive mental health screens are being developed for use at prison reception to develop effective care strategies (Senior *et al.* 2012), a possible explanation for the results of the meta-analysis may be that there is a high rate of false positives identified by ADHD screens in youths/adults compared with the diagnostic interview-only subset. Retrospective screening and interview accounts of childhood symptoms given by adult inmates were estimated to be significantly higher than those given by a current diagnoses cohort (36.4% *v.* 21.7%). It seems important that all inmates are considered for mental health screening, including ADHD, and those with positive screens be referred for a clinical assessment. What is unknown, however, is the rate of false-negative screens in this population.

The predominantly reported analysis used prevalence obtained only from diagnostic clinical interview data. This is also consistent with the diagnosis of ADHD in the community and enables a fairer comparison when comparing rates with the general population.

Consistent with prevalence and patterns of remission in the general population (Polanczyk *et al.* 2007; Young & Gudjonsson, 2008; Simon *et al.* 2009), we would expect adult prevalence to have been lower than youth prevalence; yet when applying a diagnostic interview (model 2), these were very similar (26.2% *v.* 30.1%). It has been reported that the onset of offending is at a younger age for youth with ADHD, even as young as 10 years of age (Langley *et al.* 2010; Young *et al.* 2010*b*), and perhaps these young offenders are less likely to be given a custodial sentence and/or are diverted out of the criminal justice system (e.g. to residential homes as ‘at-risk’ youths). However, an arbitrary classification was determined in the current review to stratify youth and adult offenders (i.e. if the mean/median age was above 18 years, a study was classified as an ‘adult study’). This might have led to some youth diagnoses being included as an adult diagnosis, and possibly skewed the data.

### Geographical differences

Large differences were reported between countries. However, when grouped into three regions (North America, Europe and Other countries), these differences were not significant. Europe had the highest estimated rate, followed by North America and then Other countries. These variations may be due partly to study sample sizes: 70% (seven of 10) of the diagnostic interview studies carried out in North America had sample sizes >200, compared with only 22% (two of nine) of European studies. The North American prevalence (26.9%) is closer to the prevalence obtained overall (by diagnostic interview) for offenders with youth and adult ADHD (30.1% and 26.2%, respectively). Hence, these larger samples may give an accurate estimate of prevalence.

The differential may also reflect differences in the North American and European criminal justice systems. Following the introduction of tougher laws and penalties surrounding drug crimes by the US government, the number of inmates convicted of a drug crime rose by 1300% in the USA between 1980 and 2001 (Jensen *et al.* 2004). This compares with only 12% of a Scottish prison population incarcerated for drug-related crimes (Young *et al.* 2010*b*). Nevertheless, just under half of this Scottish sample self-reported drug dependence, and hierarchical multiple regressions indicated that previous drug use was the most powerful predictor of the total extent of offending, whereas a history of ADHD symptoms was the most powerful predictor of violent crimes. Thus, US prison populations may have an over-representation of those who have been incarcerated for drug crimes, which may not necessarily be related to ‘reactive, impulsive offences' (e.g. robbery and property crimes) thought to be associated with ADHD (Retz & Rosler, 2009).

### Gender differences

Female adults (with diagnostic interview) had the lowest predicted ADHD prevalence when comparing by age and gender (22.1% *v.* 31.2%, male adults). However, female youths had similar prevalence to male youths (30.8% and 29.5%, respectively). The general population prevalence indicates a 4:1 ratio of boys to girls with ADHD (Cuffe *et al.* 2005), yet in the prison population this ratio seems much reduced. Hence, the protective mechanisms that may usually keep females out of prison (e.g. sentencing policies, family considerations) seem to be overridden for female offenders with ADHD. However, only a few data sets reporting on female prevalence were included in the ‘diagnostic interview’ analysis (*n* = 8) compared with male prevalence (*n* = 22), which may have given a restricted representation of the position. A recent study from Konstenius *et al.* (2012) sampled 56 adult incarcerated women who were assessed by diagnostic interview in a Swedish prison, and reported that 29% met DSM-IV criteria for ADHD. This study, not identified during our initial literature search, supports a similar ADHD prevalence in female adults to this meta-analysis. In the present review, male adult diagnostic interview data are the most reliable because of the quantity and hence high power available: 14752 male youth interview subjects and 1978 male adult interview subjects compared with 2623 female youth and 222 female adult subjects.

The prevalence rate of males to females in adults with ADHD (~1.5:1) is much lower than the observed ratio of 4:1 in youths with ADHD (Montejano *et al.* 2011; Willcutt, 2012; McCarthy *et al.* 2012). Male adults diagnosed by clinical interview showed a similar prevalence to youths (31.2% *v.* 30.8% female or 29.5% male). This suggests that incarcerated youths may remain in the criminal justice system because of repeat offending. Analysis of data from the Swedish National Register found that 36.6% males with ADHD were convicted of crime compared with 15.4% of females. Importantly, it was found that the use of ADHD medication reduced the risk of criminality among probands with ADHD: by 32% in men and 41% in women (Lichtenstein *et al.* 2012).

### Limitations

Two important caveats to this review should be noted. First, publication bias is always an issue in systematic reviews, but efforts to address this were made in several ways. Data were obtained from all available sources, including those from electronic databases and authors, and review papers were checked (post-2006). Bias in study selection was addressed by delivering training on how to extract data from a random sample of publications, and each researcher checked and independently reviewed a random sample of each others’ papers for data interpretation consistency. Second, factors such as the reliability of ADHD diagnoses and differences in the criminal justice system between and within countries could have contributed to the broad ranges of prevalence observed in this review. The heterogeneity of samples used is also a potential source of variability and was the reason for using a fitted model that assumed a fixed effect (equal across studies) of measurable covariates but random study effects in the meta-analysis. In addition, only those publications since 1980 and in English were included.

## Conclusions

Overall, the estimated prevalence of ADHD in incarcerated populations is 25.5% based on diagnostic clinical interviews. The ADHD prevalence was estimated to be significantly lower in studies with current diagnoses compared with retrospective diagnoses. There was a large variation in ADHD prevalence between countries, but this was no longer evident when grouped regionally. This systematic review and meta-analysis found, on average, a fivefold increased prevalence of ADHD in youth prison populations (30.1%) and a 10-fold increase in adult prison populations (26.2%) compared with published general population prevalence (3–7% and 1–5%, respectively). Female adults had a lower prevalence of ADHD, when compared by age and gender, than male adults. However, the risk for females was almost as high as that for males.

## Supplementary material

For supplementary material accompanying this paper visit http://dx.doi.org/10.1017/S0033291714000762.
